# A weather-informed decision tree framework for predicting stripe rust outbreaks in wheat

**DOI:** 10.3389/fpls.2026.1812395

**Published:** 2026-04-24

**Authors:** Shubham Anand, Sarabjot Kaur Sandhu, Barun Biswas, Parminder Singh Tak

**Affiliations:** 1 Department of Climate Change and Agricultural Meteorology, Punjab Agricultural University, Ludhiana, India; 2 Department of Plant Pathology, Punjab Agricultural University, Ludhiana, India

**Keywords:** CART analysis, decision tree, principal component analysis, real time disease assessment, stripe rust severity

## Abstract

Wheat stripe rust (caused by *Puccinia striiformis*) is highly influenced by weather conditions making weather based predictive modelling an essential tool in combating this disease. Keeping this in mind, a weather-based decision tree approach has been applied to predict stripe rust severity in Ludhiana district of Punjab. The meteorological data for the period (from 2009–10 to 2022-23) were collected from the Department of Climate Change and Agricultural Meteorology, PAU, Ludhiana. Weekly observations of yellow rust symptoms on crop leaves were recorded using modified Mannar’s scale for yellow rust of wheat. Area under disease progression curve (AUDPC) was computed for rust severity among different years. The year 2012–13 recorded the highest rust severity (100%) and AUDPC (671.3) marking it as a major epidemic year. In contrast, lowest disease severity was recorded in 2021-22 (56.75%, AUDPC 222.85) and 2022-23 (58.42%, 244.95). Correlation analysis and Principal Component Analysis (PCA) were carried out between primary and derived variables and yellow rust severity and AUDPC. Using Classification and Regression Tree (CART) analysis, decision tree models were developed which conclude that maximum stripe rust severity (79%) can occur when minimum temperature ≥ 9.1 °C and sunshine hours ≥ 9.2 h, or when mean temperature ≥ 15 °C along with humid thermal index (HTI) ≥ 2.4. Yellow rust severity remained moderate (34-39%) when Tmin < 9.1 °C, Tdew ≥ 6.2 °C and RHm ≥ 94%. Various accuracy metrices were used to compare developed decision tree models and all-weather variable model surpassed the performance than primary and derived weather variable models. Such models can be helpful in real time disease risk assessment and crop agro-advisories.

## Introduction

The global community of wheat scientists is very concerned about the on-going process of the emergence of new races of stripe rust that are virulent against cultivars currently in existence (Duplessis et al, 2011). Pathotype 46S119 is believed to have evolved from pathotype 46S103 (+vYr9) and was the first pathotype in India to have virulence to Yr9 (Prashar et al, 2007). The samples taken from across Punjab showed that the most common *Pst*, 78S84 and 46S119, had frequency variations of 75:25, 35:65, and 20:80 in the years 2010-11, 2011-12, and 2012-13, respectively (Pannu et al, 2014). The year 2014 also observed the evolution of two novel pathotypes (110S119 and 238S119) with coupled virulence to Strubes Dickkopf (YrSD, Yr25) and Suwon92xOmar (YrSU) (Gangwar et al, 2019). The pathotype 78S84, which predominated from 2005–06 to 2010-11, was not observed in nature during the two growing seasons of wheat (2017-18, 2018-19). It’s interesting to note that pathotypes 110S119 and 238S119 have gradually increased in frequency. In a recent study, it was discovered that pathotype 238S119 is more aggressive than pathotype 110S119 because it produces more urediniospores and has higher uredinal density (Singh et al., 2020).

The dynamics of wheat stripe rust (SR) is very much dependent on prevailing weather conditions making weather-based predictive modelling an essential tool for real time disease assessment. A number of plant biologists have devoted greater efforts toward studying the interactions and direct/indirect impacts of various aspects of climate on the development of plant disease epidemics. The previous studies are worth stressing the degree of effort to model the issue by including multiple meteorological parameters. At an average daily temperature of 20°C, the latent period of infection is typically 7–10 days and is temperature and moisture dependent. A leaf that appears healthy could harbour the latent yellow rust infection in extremely cold weather. The main causes of the difficulty in predicting the rate of disease development and infection transmission are the uncertainty of weather conditions and the lack of information on the levels of latent infections (Jindal et al., 2012). Newbery et al. (2016) proposed a graphical scheme to have more concise understanding of how climate, crop growth and disease models can be combined to produce projections of crop growth stages and disease incidence/severity for different climate change scenarios. Based on the weather of the same year, Malicdem and Fernandez (2015) used an Artificial Neural Network and a Support Vector Machine method to predict the occurrence and severity of rice blast disease in particular rice growth stages. They used Principal Component Analysis (PCA) to pre-process the data and identify the most crucial meteorological data. Using a variety of neural network techniques, Muthukannan et al. (2015) identified spot diseases in leaves and categorised them according to the sorts of diseased leaves. The technique of classifying diseased plant leaves by processing the set of shape and texture features from the afflicted leaf picture utilising Radial Basis Function Networks (RBF), Learning Vector Quantization (LVQ), and Feed Forward Neural Networks (FFNN). Another strategy used by Sannakki et al. (2013) was combining the crop forecasting regression model with weather forecasting using an artificial neural network using a modified K-Nearest-Neighbour validation. Based on predicted meteorological information including temperature and humidity, the authors created a system that could anticipate grape powdery mildew. A system based on the Internet of Things that can monitor and manage wheat diseases, weeds, and pests has been proposed by Zhang et al. (2014). The expert system collects historical information about weeds, pests, and diseases that affect wheat as well as a large number of digital photos of these issues in wheat fields.

Basic studies on temperature, relative humidity, rainfall, sunshine hours for yellow rust have been documented in earlier research. Temperature and light have been observed as critical abiotic factors for urediniospore germination and infection. Research revealed that germination thrives between 10 °C and 28 °C, with good germ tube growth at 15-20 °C while nighttime temperatures of 10-12 °C provide ideal conditions for causing infection and latent periods of 10–15 days (Ellison et al., 1990; Line, 2002). If optimum temperature light temperature combination occurs, *Puccinia striiformis* may proliferate unlike other fungi (Schroder and Hassebrauk, 1964). A combination of 15 °C temperature, pH of 7 and light intensity of 1250 lux promote germination of urediniospores of yellow rust. So, this all shows the role of abiotic factors on yellow rust. But very limited work has been done to quantify the severity of yellow rust that would occur if certain weather conditions prevailed. Previous studies have examined the relationship between weather variables and stripe rust occurrence. However, limited work has focused on explicitly quantifying the expected level of disease severity under specific weather conditions. In this context, the present study employs CART analysis to predict severity levels based on defined weather thresholds, thereby enhancing its utility for disease forewarning. This would help in knowing potential severity of yellow rust under certain weather conditions. This can serve as an excellent practical tool for early warning systems and timely disease management.

## Materials and methods

### Study area

The present study was conducted in Ludhiana, Punjab, India (30°54′ N latitude, 75°48′ E longitude; 247 m above mean sea level), situated at the centre of India’s trans-Gangetic plain agroclimatic zone. This location is considered as a representative site for the edaphic and climatic conditions of India’s wheat-basket. The climate of the study area is classified as subtropical and semi-arid type (‘BSh’ as per Köppen’s climate classification) (Chen and Chen, 2013). The area is characterized by annual average maximum and minimum temperatures of 29.8 °C and 16.9 °C, respectively. During December and January, the minimum temperature occasionally falls below 0 °C for a few days, resulting in ground frost. In contrast, the maximum temperature often exceeds 45 °C during the summer months of May and June. The study area receives an annual average rainfall of 761 mm, of which 68% occurs during the monsoon season (June–September). The wheat growing winter (*rabi*) season receives about 16% of annual normal rainfall largely due to western disturbances. Increasing variability in temperature and rainfall in recent decades, both inter-seasonal and inter-annual, has elevated the risk of foliar diseases in this major wheat-growing region.

### Experimental details and disease assessments

Field experiments were conducted at the Research Farm of Punjab Agricultural University, Ludhiana, Punjab, India, during the rabi seasons from 2009–10 to 2022–23. The rabi seasons of 2017–18 and 2020–21 were excluded due to insufficient disease observations, caused by the concurrent occurrence of multiple foliar diseases and the COVID-19 lockdown, respectively. Two widely adopted wheat varieties of Punjab i.e. PBW 343 (up to 2012–13) and HD 2967 were grown to evaluate the incidence, progress, and severity of stripe rust. The crop was sown during the normal sowing window (first fortnight of November) in each season. To ensure continuity of disease data, artificial inoculation was carried out in December each year.

Stripe rust intensity and disease progress were assessed using the modified Cobb’s scale (Peterson et al., 1948) (**Table 1**). Subsequently, the stripe rust severity (SR) was calculated from the periodic intensity data using the following formula:

**Table 1:** Modified Cobb’s scale for stripe rust intensity.

Code	Percent of infection
5	Up to 5 per cent leaf area infected
10	Up to 10 per cent leaf area infected
20	Up to 20 per cent leaf area infected
50	Up to 50 per cent leaf area infected
75	Up to 75 per cent leaf area infected
100	Up to 100 per cent leaf area infected


$$\text{Disease severity}(\%)=\frac{\sum \text{Number of infected leaves}\times \text{Scale}}{\text{Total number of leaves}\times \text{Maximum grade}}\times 100$$

Additionally, the area under the disease progress curve (AUDPC) was calculated using the Wilcoxson et al. (1975) method to compare overall seasonal variability of SR severity over the study period. The AUDPC was quantified as follows:


$$\text{AUDPC}=\sum_{\text{i}=1}^{\text{n}=1}(\frac{\text{Xi}+(\text{Xi}+1)}{2})\times ({\text{t}}_{\text{i}}+1-{\text{t}}_{\text{i}})$$

Where,

X*i* = Disease severity on day *i*
t*i*= time in days between *i* and date *i* + 1
n = number of observations

### Primary and derived meteorological parameters

Daily meteorological data for the respective crop growth seasons were collected from the Agrometeorological Observatory, located approximately 100 m from the experimental plots. The primary parameters, recorded at the observatory, included maximum air temperature (Tmax), minimum air temperature (Tmin), morning relative humidity (RHm), evening relative humidity (RHe), sunshine hours (SSh) and rainfall (RF).

Derived meteorological parameters were empirically calculated from the primary data to represent additional atmospheric conditions influencing disease development. The mean air temperature (Tme) and mean relative humidity (RHme) were obtained as the arithmetic means of the respective maximum and minimum values. The dew point temperature (Tdew) and the humid-thermal index (HTI) were calculated using the following relations:


$$\text{Tdew}=\text{Tme}-\frac{100-RHme}{5}$$

$$\text{HTI}=\frac{RHe}{Tmax}$$

Subsequently, the daily primary and derived meteorological parameters were averaged over each disease observation interval of the respective season to explore inter-relationships with wheat rust severity and to develop a suitable predictive model.

### Assessment of influence of meteorological parameters on stripe rust

The average meteorological parameters during the disease initiation, progression and end phase were explored by calculating their range (minimum - maximum) and mean values. The inter-relationships of SR and AUDPC with individual meteorological parameters were examined using Pearson’s correlation and simple linear regression. These statistical analyses were essential for identifying the influential meteorological factors responsible for wheat rust infection and disease progression.

Subsequently, Principal Component Analysis (PCA) was performed to further explore the inter-relationships between disease severity and meteorological parameters, including both the direction and degree of association. In this analysis, all meteorological parameters were considered as primary input variables, while rust severity indices (SR and AUDPC) were treated as quantitative supplementary variables. Scaled values were used to overcome unit mismatches among variables. PCA helped to address the limitations of linear relationship analyses using correlation and regression (e.g., multicollinearity) and examined the role of different primary and derived meteorological conditions in determining wheat rust severity. The exploratory results of the PCA are presented through a scree plot, a correlation-variable contribution bi-plot and a combined contribution plot of different meteorological variables in 1^st^ and 2^nd^ principal components.

### Development of regression-based decision tree model

A regression-based decision tree approach was employed to develop a weather-informed framework for predicting weekly stripe rust severity in wheat. The model was implemented using the Classification and Regression Tree (CART) machine learning algorithm. To ensure robust model development and independent validation, the dataset was partitioned chronologically. The first 10 seasons were used as the training dataset for model development, while the most recent two seasons (2021–22 and 2022-23) were reserved as an independent test dataset for model evaluation. This temporal split ensured that the model was evaluated under unseen seasonal conditions, mimicking real-world forecasting scenarios.

Regression tree models were developed using the CART algorithm with stripe rust severity (SR, %) as the response variable and meteorological parameters as predictor variables. Three different predictor sets (primary, derived and all meteorological parameters) were evaluated to identify the most informative weather inputs.

Initial tree models were grown without constraints to allow maximal splitting. To avoid overfitting and improve generalization, tree complexity was controlled using cost-complexity pruning. A complexity parameter threshold of 0.01 was imposed, which penalizes additional splits unless they significantly reduce model error. Furthermore, 10-fold cross-validation was employed during model training to estimate the cross-validated error associated with different tree sizes. The optimal tree size was selected based on the cross-validation results using the complexity parameter table and corresponding plots. The final model was chosen by identifying the cp value that minimized the cross-validated relative error, while maintaining model parsimony to avoid overfitting.

The predictive performance of the developed models was evaluated using an independent test dataset. Model accuracy and robustness were assessed through multiple statistical indicators, including Root Mean Square Error (RMSE), Relative Root Mean Square Error (RRSE, %), Pearson’s correlation coefficient (r), Mean Absolute Error (MAE), modified index of agreement (md), and modified Nash–Sutcliffe Efficiency (mNSE). These metrics collectively quantify both error magnitude and goodness-of-fit between observed and predicted stripe rust severity values. Models developed using different predictor sets (all variables, primary variables, and derived variables) were compared based on their performance on the test dataset. The final decision tree model for stripe rust prediction was selected considering a balance between predictive accuracy, model simplicity, and interpretability of decision rules.

### Statistical software

All statistical data analysis and model development in the present study was executed using the R Statistical Language ver. 4.4 (R Core Team, 2024). The functions from base R packages were utilized primary data input and management. Different functions available in ‘*tidyverse*’ package was used for data manipulation and summary statistics calculations. The AUDPC values were computed by utilizing the function available in ‘*agricolae*’ package. The panel plots representing results of correlation and regression analysis was created using a new function developed based on ‘*GGally*’ and ‘*ggpmisc*’ packages. The regression-based tree models were developed using the CART based machine learning algorithm and functions available in ‘*rpart*’ R package.

## Results

### Rust severity and AUDPC

**Figure 1:**
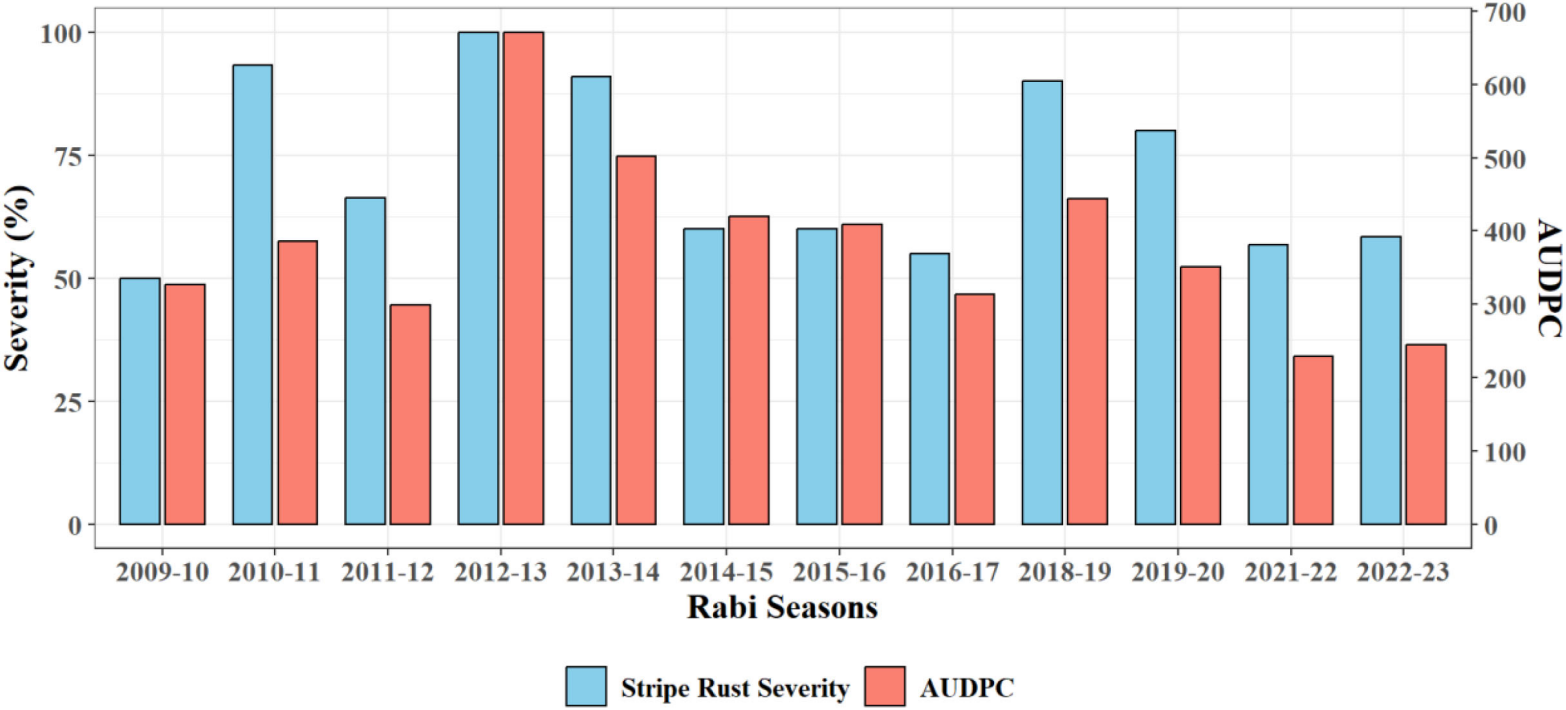
Seasonal variability in maximum stripe rust severity (%) and cumulative AUDPC during the study period.

The year 2012–13 recorded the highest rust severity (100%) and AUDPC (671.3) marking it as a major epidemic year (**Figure 1**). Similarly, 2013–14 and 2010–11 also showed high rust severity (91% and 93.33%) with AUDPC values of 501.6 and 386.55, respectively. In contrast, lowest disease severity was recorded in 2021-22 (56.75%, AUDPC 222.85) and 2022-23 (58.42%, 244.95). Overall, years with higher AUDPC generally coincided with high rust severity.

### Meteorological parameters during disease period

During disease initiation phase, the mean maximum temperature varied notably across the different years. The lowest Tmax was recorded in 2019-20 (13.4 °C) followed by 2021-22 (16 °C), 2010-11 (16.2 °C), 2014-15 (17.1 °C), 2013-14 (17.5 °C), 2012-13 (17 °C) and 2016-17 (17.7 °C). A gradual increase was observed in subsequent years with 2011-12 (18.8 °C), 2018-19 (19.2 °C) and 2022-23 (19.3 °C) (**Tables 2**–**5**). Higher temperatures were recorded during 2015-16 (20.5 °C) and 2009-10 (19.7 °C). The average Tmin was observed low in years 2011-12 (4.7 °C) followed by 2018-19 (5.1 °C), 2010-11 (5.4) and 2012-13 (5.9 °C) but higher Tmin was recorded in years 2022-23 (6.8 °C), 2014-15 (7 °C), 2013-14 (7.3) and 2021-22 (7.7 °C). Morning relatively humidity was higher (above 90%) during all the study years with highest in case of year 2011-12 (98.6%) followed by 2010-11 (97%), 2009-10 (95.6%) and 2013-14 (95.4%). The evening relative humidity was below 75 percent during all years under study with highest in case of year 2014-15 (74%) and lowest in case of 2015-16 (41.3%). Increased relative humidity driven by rain also has a direct impact on uredospore formation. The sunshine hours/day were recorded higher in year 2011-12 (6.1 h/day) followed by 2015-16 (5.5 h/day) and 2022-23 (5.13 h/day) at disease initiation. Total rainfall was recorded more than 30 mm during 2013-14 (56.5 mm) following 2018-19 (50.4 mm), 2021-22 (44.2 mm), 2014-15 (42.6 mm), 2022-23 (32.1 mm) and 2012-13 (30.8 mm). Dew point temperature was higher in year 2013-14 (8.3 °C), 2021-22 (8.3 °C) and 2014-15 (8.4 °C) and lower in years 2018-19 (5.9 °C), 2019-20 (6.2 °C) and 2015-16 (6.8 °C). HTI was high in 2019-20 (5.5), 2021-22 (5.43), 2010-11 (4.58) and 2014-15 (4.42) and low in years 2015-16 (2.01), 2018-19 (2.37), 2022-23 (2.58) and 2011-12 (2.93).

**Table 2:** Meteorological parameters during disease initiation, progression and end phase during 2009-10, 2010–11 and 2012-13.

Year	Phase	Primary weather variables						Derived weather variables			
		Tmax (°C)	Tmin (°C)	RHm (%)	RHe (%)	SSh (h/day)	Total RF (mm)	Tme (°C)	RHme (%)	Tdew (°C)	HTI
2009-10	Initiation	14.2-23.4 (19.7)	3.8-8.8 (6.6)	92-99 (95.6)	41-81 (52.8)	1.4-6.1 (4.6)	18	10.7-15.7 (13.2)	67-89 (75.4)	6.7-9.2 (8.0)	1.75-5.7 (3.32)
	Progression	11.9-21.1 (17.6)	5.5-10.1 (7.1)	94-100 (97.6)	53-81 (65.2)	1.2-7.7 (4.0)	25	9.2-15.4 (12.4)	74-91 (81.8)	7.3-10.3 (8.7)	2.51-6.81 (4.25)
	End	21.5-34.2 (27.3)	7.2-17.1 (12.6)	90-96 (93.9)	36-48 (43.7)	7.9-10.2 (9.1)	1	14.4-25.7 (20.1)	65-72 (68.8)	8.7-18.6 (14.4)	1.05-2.23 (1.58)
2010-11	Initiation	11.0-19.9 (16.2)	4.4-6.2 (5.4)	95-99 (97.0)	56-84 (67.8)	1.3-5.7 (3.1)	23.0	8.6-12.3 (10.8)	77-90 (82.8)	6.5-8.3 (7.3)	2.81-7.64 (4.58)
	Progression	16.4-22.1 (19.9)	4.1-6.8 (6.5)	92-97 (95.3)	51-66 (58.8)	5.2-7.9 (6.3)	8.2	10.6-14.5 (13.3)	74-81 (78.0)	6.4-10.1 (8.8)	2.62-4.02 (3.23)
	End	19.7-24.8 (21.5)	8.7-10.7 (10.0)	92-97 (94.3)	55-74 (66.3)	3.9-10.1 (5.8)	42.5	14.9-17.3 (15.8)	75-83 (80.3)	11.3-12.3 (11.8)	2.22-3.76 (3.13)
2011-12	Initiation	16.6-20.9 (18.8)	3.0-7.3 (4.7)	97-99 (98.6)	46-72 (54.0)	2.1-8.0 (6.1)	14.6	9.8-13.5 (11.8)	73-85 (76.6)	5.3-9.1 (7.1)	2.29-4.21 (2.93)
	Progression	14.3-18.4 (16.1)	3.0-7.7 (6.2)	93-99 (95.4)	47-83 (68.2)	2.0-8.6 (4.5)	52.6	9.8-12.2 (11.3)	70-89 (80.2)	5.3-9.1 (7.6)	2.55-5.8 (3.94)
	End	17.8-24.5 (21.5)	4.2-10.1 (7.3)	89-96 (91.3)	41-52 (46.6)	5.8-8.9 (7.5)	4.6	11.0-17.3 (14.4)	66-71 (68.4)	5.0-10.4 (8.2)	1.67-2.58 (2.26)

**Table 3:** Meteorological parameters during disease initiation, progression and end phase during 2012-13, 2013–14 and 2014-15.

Year	Phase	Primary weather variables						Derived weather variables			
		Tmax (°C)	Tmin (°C)	RHm (%)	RHe (%)	SSh (h/day)	Total RF (mm)	Tme (°C)	RHme (%)	Tdew (°C)	HTI
2012-13	Initiation	10.5-20.9 (17.6)	4.7-8.1 (5.9)	90-98 (94.6)	49-75 (60.6)	0.2-8.4 (4.8)	30.8	7.6-14.5 (11.3)	72-83 (78.0)	4.1-10.4 (7.2)	2.51-7.14 (3.81)
	Progression	19.8-28.7 (22.6)	7.4-12.9 (10.1)	94-99 (96.6)	50-73 (62.0)	5.3-10.0 (7.8)	71.2	13.6-20.8 (16.4)	72-86 (77.8)	10.1-15.2 (12.3)	1.74-3.65 (2.96)
	End	27.2-32.0 (29.0)	12.8-15.3 (14.5)	81-95 (90.3)	25-51 (42.8)	9.2-11.1 (9.8)	35.6	20.0-23.7 (21.8)	53-73 (66.8)	14.3-16.0 (15.1)	0.78-1.88 (1.50)
2013-14	Initiation	15.7-18.9 (17.5)	3.8-9.7 (7.3)	93-98 (95.4)	51-75 (63.2)	2.5-6.1 (4.3)	56.5	10.3-14.1 (12.4)	72-86 (79.4)	4.9-10.9 (8.3)	2.87-4.78 (3.63)
	Progression	18.3-23.8 (20.7)	6.7-11.4 (8.8)	88-95 (92.2)	57-64 (60.6)	5-11.7 (7.3)	42.5	12.5-17.6 (14.7)	73-80 (76.6)	7.9-12.1 (10.0)	2.39-3.5 (2.97)
	End	26.2-28.8 (27.3)	12.6-15.1 (13.9)	84-93 (88.8)	43-52 (47.3)	7.3-10.1 (8.9)	32.0	19.5-22.0 (20.9)	64-72 (68.3)	13.9-14.7 (14.3)	1.49-1.98 (1.74)
2014-15	Initiation	12.5-25.1 (17.1)	5.2-8.2 (7.0)	92-97 (94.7)	60-82 (74.0)	0.9-7.9 (3.2)	42.6	9.3-16.4 (11.9)	68-90 (81.6)	7.2-9.9 (8.4)	1.67-6.48 (4.42)
	Progression	13.3-20.9 (17.0)	6.2-7.7 (7.1)	92-97 (95.2)	59-80 (69.2)	0.3-7.4 (4.5)	37.0	10.3-14.1 (12.1)	77-88 (82.0)	7.8-9.5 (8.5)	2.82-6.02 (4.67)
	End	20.3-24.1 (22.9)	9.2-14.5 (11.6)	93-94 (93.4)	61-77 (66.2)	3.7-8.3 (6.4)	96.6	15.5-19.0 (17.5)	78-86 (80.0)	11.4-16.1 (13.2)	2.59-3.3 (2.90)

**Table 4:** Meteorological parameters during disease initiation, progression and end phase during 2015-16, 2016–17 and 2018-19.

Year	Phase	Primary weather variables						Derived weather variables			
		Tmax (°C)	Tmin (°C)	RHm (%)	RHe (%)	SSh (h/day)	Total RF (mm)	Tme (°C)	RHme (%)	Tdew (°C)	HTI
2015-16	Initiation	20.0-21.1 (20.5)	4.9-7.3 (5.8)	94-95 (94.7)	37-49 (41.3)	2.9-7.5 (5.5)	0	12.5-14.2 (13.2)	66-72 (68.3)	5.8-8.6 (6.8)	1.81-2.32 (2.01)
	Progression	13.0-21.4 (17.6)	5.2-8.2 (7.4)	89-97 (93.4)	48-77 (61.0)	0.2-6.5 (3.9)	20.2	9.7-14.8 (12.5)	69-86 (78.8)	6.3-8.8 (7.6)	2.24-5.92 (3.74)
	End	22.1-28.1 (25.1)	7.5-14.5 (12.2)	89-94 (91.6)	41-56 (47.8)	6.5-8.1 (7.6)	45.9	14.8-20.6 (18.6)	65-74 (70.0)	7.8-14.3 (13.0)	1.49-2.31 (1.92)
2016-17	Initiation	16.0-20.9 (17.7)	3.5-9.7 (6.5)	94-95 (94.7)	43-64 (54.7)	3.3-6.5 (4.8)	5.6	9.9-15.3 (12.1)	69-79 (74.7)	3.7-10.5 (7.0)	2.64-4.00 (3.12)
	Progression	19.5-20.7 (20.0)	8.2-10.3 (9.1)	92-96 (93.7)	53-64 (60.3)	3.6-6.0 (4.7)	47.2	14.3-14.9 (14.6)	73-80 (77.3)	9.0-10.6 (10.0)	2.56-3.28 (3.02)
	End	24.3-25.5 (24.9)	9.5-10.6 (9.9)	89-90 (89.7)	34-44 (39.0)	8.1-9.6 (8.8)	0	17.0-17.7 (17.4)	62-67 (64.3)	9.9-10.5 (10.3)	1.33-1.81 (1.57)
2018-19	Initiation	18.2-20.3 (19.2)	2.8-6.7 (5.1)	90-95 (92.2)	39-53 (45.2)	2.2-6.3 (4.6)	50.4	10.6-12.9 (12.2)	66-73 (69.0)	4.3-7.0 (5.9)	1.92-2.91 (2.37)
	Progression	17.2-19.8 (19.0)	5.9-10.6 (7.8)	91-94 (92.5)	52-62 (56.5)	2.4-7.6 (4.9)	100.4	11.6-15.2 (13.3)	73-77 (75.0)	6.3-10.5 (8.2)	2.74-3.13 (2.99)
	End	20.0-24.6 (22.1)	9.6-10.7 (10.2)	87-91 (89.3)	45-58 (52.0)	6.7-8.2 (7.3)	18.2	14.8-17.7 (16.2)	68-75 (71.0)	9.2-11.2 (10.3)	1.83-2.75 (2.38)

**Table 5:** Meteorological parameters during disease initiation, progression and end phase during 2019-20, 2021–22 and 2022-23.

Year	Phase	Primary weather variables						Derived weather variables			
		Tmax (°C)	Tmin (°C)	RHm (%)	RHe (%)	SSh (h/day)	Total RF (mm)	Tme (°C)	RHme (%)	Tdew (°C)	HTI
2019-20	Initiation	10.3-16.3 (13.4)	5.4-8.1 (6.4)	89-94 (92)	63-75 (70.7)	0.9-2.8 (1.5)	13.4	7.9-11 (9.9)	78-84 (81.3)	4.3-7.7 (6.2)	3.87-7.28 (5.5)
	Progression	15.1-18.3 (16.5)	6.2-7.4 (6.9)	93 (93)	55-69 (63.7)	3.8-6.3 (4.7)	26.4	11.1-12.3 (11.7)	74-81 (78.3)	7.1-7.8 (7.4)	3.01-4.57 (3.9)
	End	17.5-23.0 (19.8)	4.9-7.8 (6.1)	93-95 (94.0)	45-59 (51.0)	7.2-9.7 (8.3)	0	11.6-15.4 (13.0)	69-77 (72.7)	6.2-9.2 (7.5)	1.96-3.37 (2.6)
2021-22	Initiation	14.5-17.5 (16.0)	7.4-8.0 (7.7)	94-96 (95.0)	62-76 (69.0)	2.5-4.4 (3.45)	44.2	11.3-13.9 (12.6)	71-86 (78.5)	8.1-8.5 (8.3)	2.46-8.4 (5.43)
	Progression	19.9-23.4 (21.65)	7.2-7.8 (7.5)	92-94 (93.0)	42-49 (45.5)	8.4-9.0 (8.7)	0	13.9-15.3 (14.6)	68-74 (71.0)	8.9-10.3 (9.6)	7.3-9.0 (8.15)
	End	22.0-31.8 (25.1)	10.2-17.3 (13.3)	85-91 (89.0)	41-58 (54.0)	1.75-2.59 (2.27)	13.3	15.7-24.6 (19.15)	63-75 (68.75)	9.4-18.1 (13.0)	1.42-2.59 (2.0)
2022-23	Initiation	18.1-20.3 (19.3)	4.3-8.6 (6.8)	89-93 (90.7)	37-57 (50.0)	2.04-6.7 (5.13)	32.1	11.2-14.5 (13.07)	63-75 (70.3)	3.8-9.5 (7.1)	2.04-2.89 (2.58)
	Progression	23.0-27.5 (25.3)	9.2-12.1 (10.4)	87-90 (88.7)	38-45 (42.0)	6.9-8.4 (7.7)	0	16.5-19.8 (17.73)	63-67 (65.3)	9.4-13.1 (11.0)	1.54-1.96 (1.69)
	End	26.8-28.5 (27.7)	13.6-13.7 (13.7)	88-89 (88.5)	40-43 (41.5)	8.1-10.3 (9.2)	2.8	20.3-21.1 (20.7)	65-66 (65.5)	13.4-14.0 (13.7)	1.40-1.60 (1.5)

During disease progression phase, Tmax was recorded higher in year 2013-14 (20.7 °C), 2021-22 (21.7 °C), 2012-13 (22.6 °C) and 2022-23 (25.3 °C) whereas low Tmax was recorded in 2011-12 (16.1 °C), 2019-20 (16.5 °C) and 2014-15 (17.0 °C). Tmin was recorded lower in years 2011-12 (6.2 °C) and 2010-11 (6.5 °C) but higher in years 2012-13 (10.1 °C) and 2022-23 (10.4 °C). RHm remained higher than 90% during all the years under study except in year 2022-23 (88.7%). RHe was below 70% during all years under study with lowest RHe during 2022-23 (42%) and 2021-22 (45.5%). Higher sunshine hours/day were recorded during 2022-23 (7.7 h/day), 2012-13 (7.8 h/day) and 2021-22 (8.7 h/day). The years 2021–22 and 2022–23 received no rainfall during disease progression phase while more than 50 mm rainfall was received during years 2011-12 (52.6 mm), 2012-13 (71.2 mm) and 2018-19 (100.4 mm). High light intensities would therefore operate to delay germination during brief wetness periods and thereby prevent urediniospores wastage. The dew point temperature was higher in years 2012-13 (12.3 °C), 2022-23 (11 °C) and 2021-22 (9.6 °C) and lowest in year 2019-20 (7.4 °C) followed by 2011-12 (7.6 °C) and 2015-16 (7.6 °C). HTI was highest during 2021-22 (8.15) and lowest during 2022-23 (1.69) and 2012-13 (2.96).

During disease end phase, higher Tmax was recorded during 2012-13 (29 °C), 2022-23 (27.7 °C) and 2013-14 (27.3 °C) while it was lower in year 2019-20 (19.8 °C), 2010-11 (21.5 °C) and 2011-12 (21.5 °C). Tmin was lowest during year 2012-23 (14.5 °C) followed by 2013-14 (13.9 °C), 2022-23 (13.7 °C) and 2021-22 (13.3 °C). Under Indian conditions, the inoculum of yellow rust cannot survive in plains because to the extremely high temperatures experienced in May and June. For the *Pst* uredospores to survive, temperature is crucial. The growth and spread of diseases as well as the process of infection are all greatly influenced by temperature. Given that yellow rust is a polycyclic disease, meteorological factors like temperature can either underplay the disease’s effects or cause epidemics to break out (Brown et al., 2001). RHm was generally higher than 88% during most of the years under study with highest in year 2010-11 (94.3%). RHe was generally lower than 67% with lowest during 2016-17 (39%) followed by 2022-23 (41.5%) and 2012-13 (42.8%). Sunshine hours/day were maximum during year 2012-13 (9.8 h/day) followed by 2022-23 (9.2 h/day), 2009-10 (9.1 h/day) and 2013-14 (8.9 h/day). The years 2016–17 and 2019–20 recorded no rainfall durinh disease end phase while the years 2012-13, 2010-11, 2015–16 and 2014–15 recorded 35.6 mm, 42.5 mm, 45.9 mm and 96.6 mm, respectively. Highest Tdew was recorded in year 2012-13 (15.1 °C) followed by 2009-10 (14.4 °C) and 2013-14 (14.3 °C). Lowest THI was observed during 2012-13 (1.5) and 2022-23 (1.5) while higher HTI was observed during 2010-11 (3.13).

### Correlation and regression analyses

**Figure 2:**
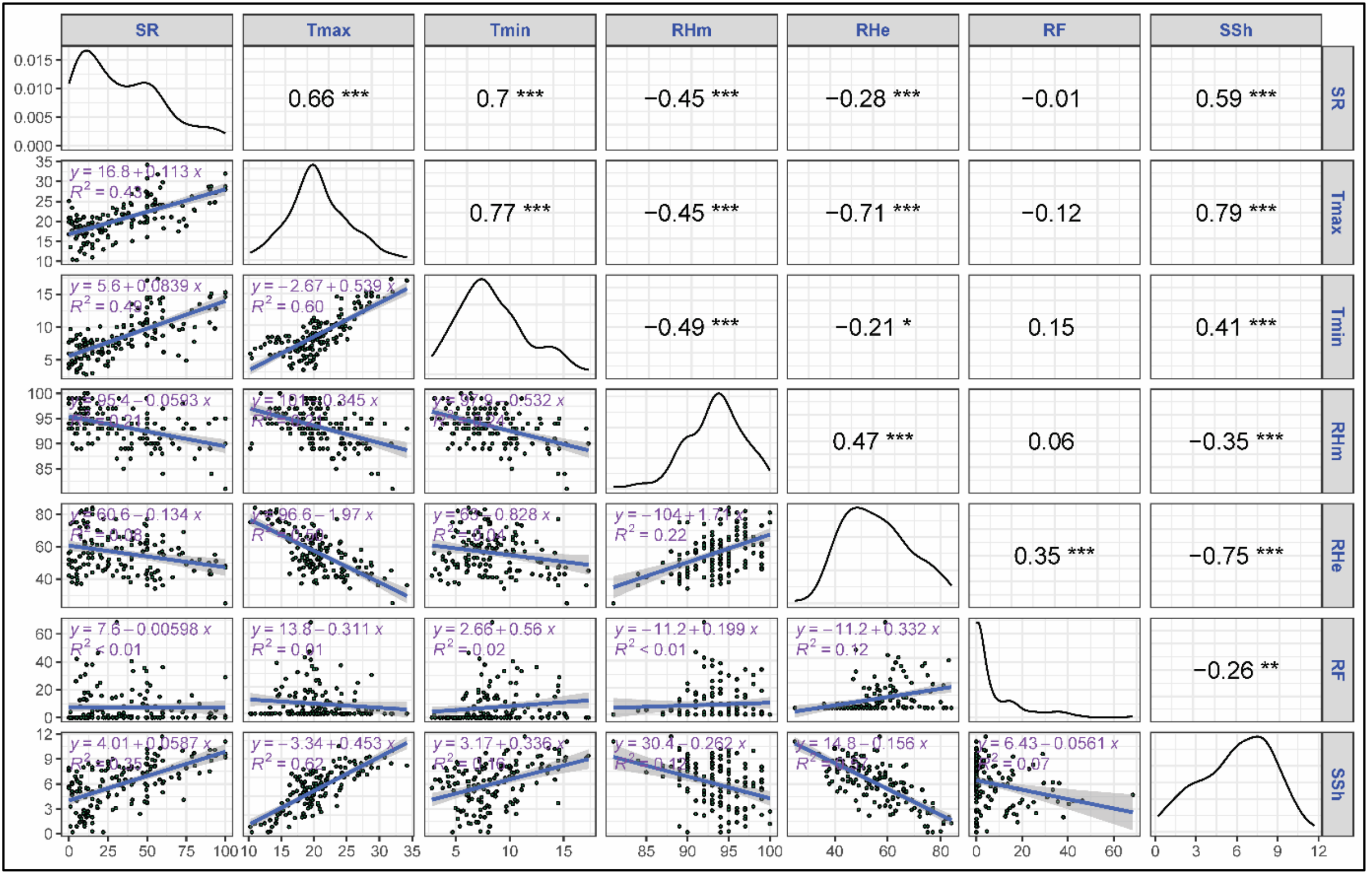
Correlation and regression: Severity vs primary weather variables. The symbols *, **, *** indicate significance levels at p < 0.05, p < 0.01, and p < 0.001, respectively.

**Figure 3:**
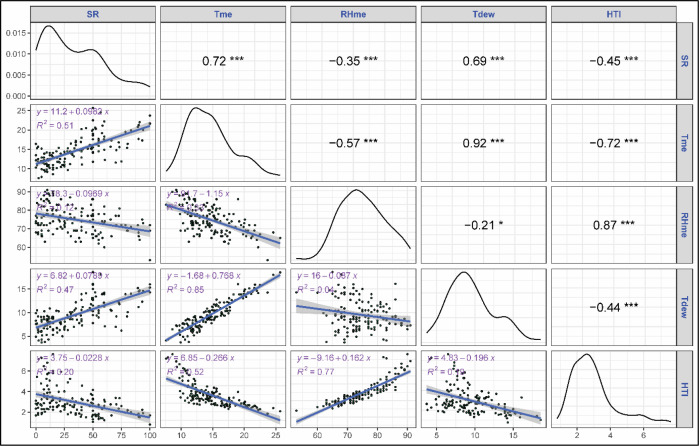
Correlation and regression: Severity vs derived weather variables. The symbols *, *** indicate significance levels at p < 0.05 and p < 0.001, respectively.

**Figure 4:**
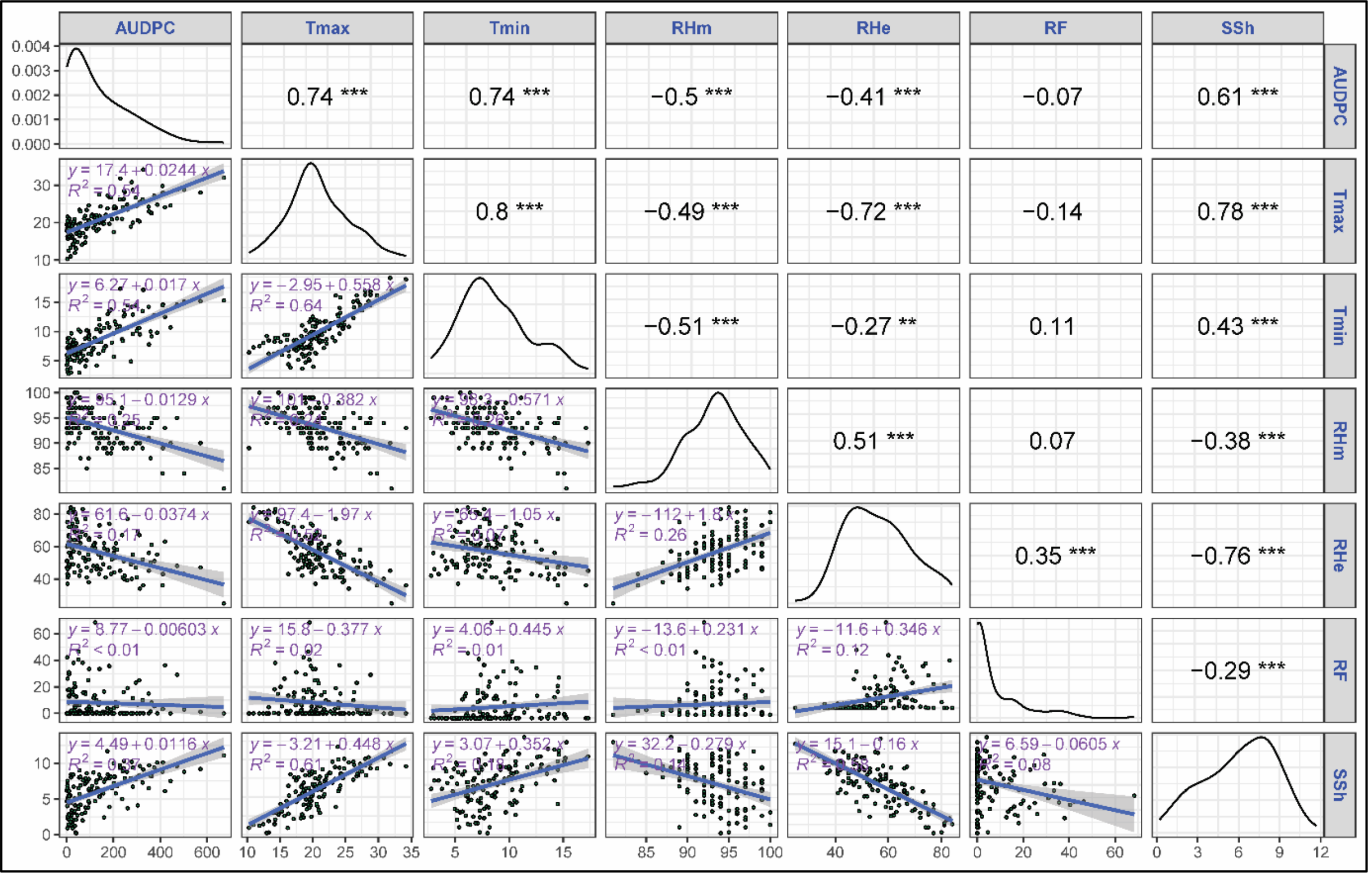
Correlation and regression: AUDPC vs primary weather variables. The symbols **, *** indicate significance levels at p < 0.01 and p < 0.001, respectively.

**Figure 5:**
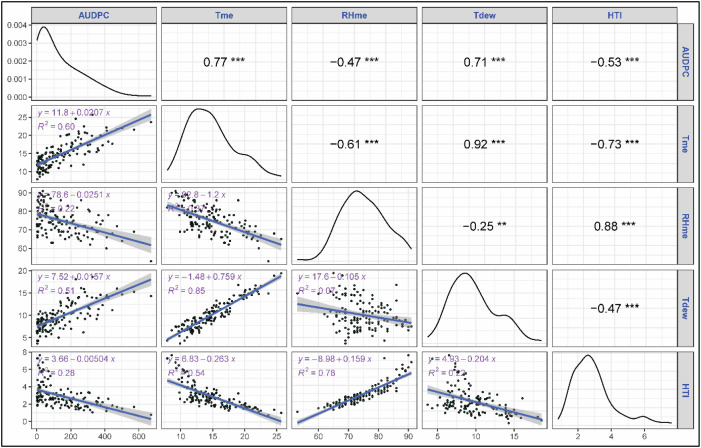
Correlation and regression: AUDPC vs derived weather variables. The symbols **, *** indicate significance levels at p < 0.01 and p < 0.001, respectively.

The correlation and regression analyses revealed key relationships between stripe rust severity (SR) and meteorological parameters. Among all variables, both maximum (Tmax) and minimum (Tmin) temperatures showed a strong positive correlation with strong positive correlation with stripe rust severity, with correlation coefficients of 0.66 and 0.70, respectively, and were highly significant (p<0.001) (**Figure 2**). Tmax and Tmin explained 43 and 49% of variation in severity as proven by regression fits. Morning and evening relative humidity showed a significant negative correlation with rust severity (r = -0.45, -0.28, p<0.001) though their regression exhibited a weaker fit with R^2^ = 0.21 and 0.08. Sunshine hours exhibited significant positive correlation with SR (r = 0.59, p<0.001) with moderate regression fit (R^2^ = 0.35). Rainfall didn’t show any significant correlation with rust severity (r = -0.01, ns). Among the derived variables mean temperature exhibited a strong positive correlation with rust severity (r= 0.72, p<0.001) with a regression coefficient explaining 51% of the variation in SR (**Figure 3**). Likewise, dew point temperature exhibited a significant positive correlation with SR (r= 0.69, p<0.001) and a good regression fir (R^2^ = 0.47). RHme and HTI showed a significant negative correlation (r= -0.35, -0.45 p<0.001) with rust severity. Both Tmax and Tmin showed a strong positive correlation (r= 0.74, 0.74) with AUDPC and good regression fits (R^2^ = 0.54, 0.54) (**Figure 4**). Sunshine hours also exhibited positive correlation (r = 0.61, p<0.001) with AUDPC while morning and evening relative humidities exhibites significant negative coreelation (r = -0.50 and -0.41, p < 0.001). Among the derived variables, Tmean and Tdew showed significant positive correlation (r = 0.77 and 0.71, p<0.001) and good regression fits with R^2^ = 0.60 and 0.51, respectively (**Figure 5**). On the other hand, RHme and HTI exhibited significant negative correlation (r = -0.53 and -0.47) with AUDPC.

### Principal component analysis

**Figure 6:**
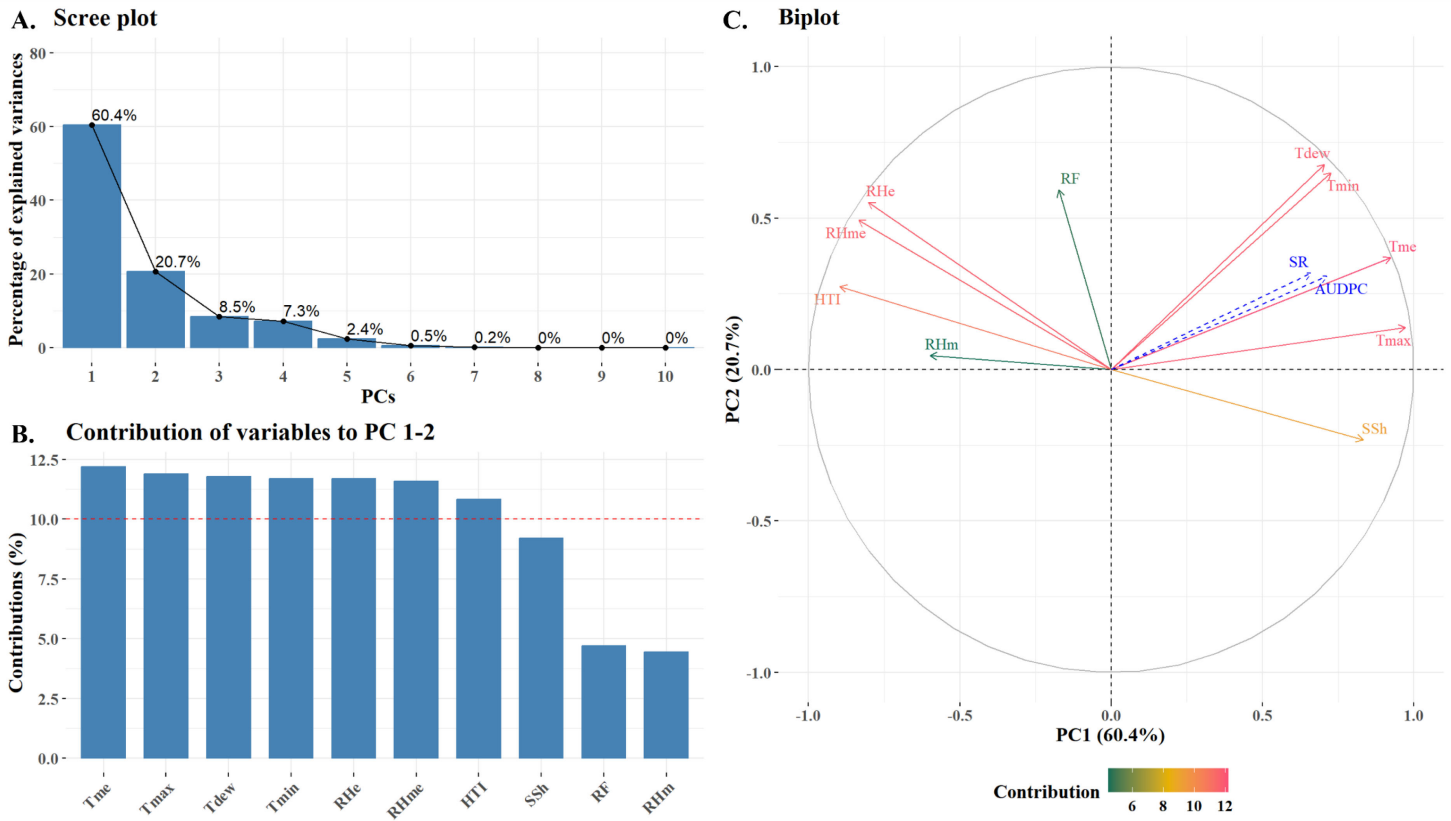
Principal Component Analysis: Scree plot **(A)**, Contribution of variables to PC 1-2 **(B)** and Biplot **(C)**.

The PCA was conducted to explore the relationship between stripe rust severity, AUDPC and weather variables. The scree plot (**Figure 6A**) showed that the first two principal components (PC_1_ and PC_2_) together explain 81.1% of the total variance (60.4% by PC_1_ and 20.7% by PC_2_). The bar plot (**Figure 6B**) displayed variable contributions to these components with Tme, Tmax, Tdew, Tmin, RHe, RHme and HTI contributing most strongly each exceeding the threshold of average expected contribution (10%). The biplot (**Figure 6C**) illustrates how variables relate to PC_1_ and PC_2_. Stripe rust severity and AUDPC were found to be positively correlated with Tmax, Tmin, Tme and Tdew while RHm, RHe and RHme, RF, HTI and Ssh were negatively correlated with both stripe rust severity and AUDPC. Gill et al. (2012) conducted correlation and regression analysis using 7 years (2004-11) of data to identify the meteorological factors influencing the development of stripe rust disease in wheat. The results indicated a positive correlation between maximum temperature in the 48^th^ and 49^th^ standard meteorological weeks (SMWs) and disease occurrence. Additionally, a negative relationship was found between sunshine duration in the 49^th^ to 50^th^ SMWs and the disease. Whereas, minimum temperature, rainfall, and relative humidity during the 2^nd^ and 3^rd^ SMWs were positively correlated with disease severity.

### CART analysis

**Figure 7:**
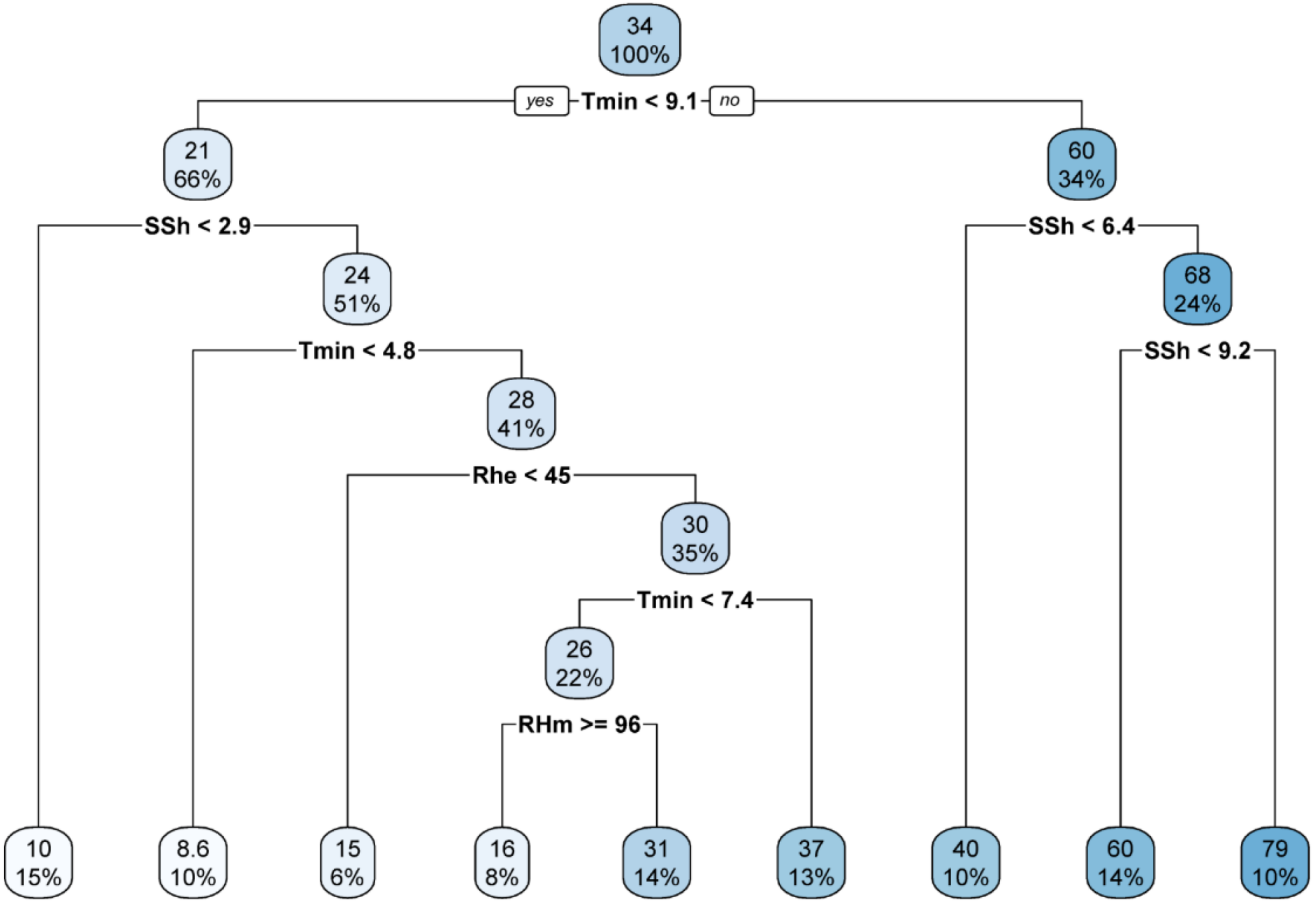
Decision tree with primary weather variable data.

**Figure 8:**
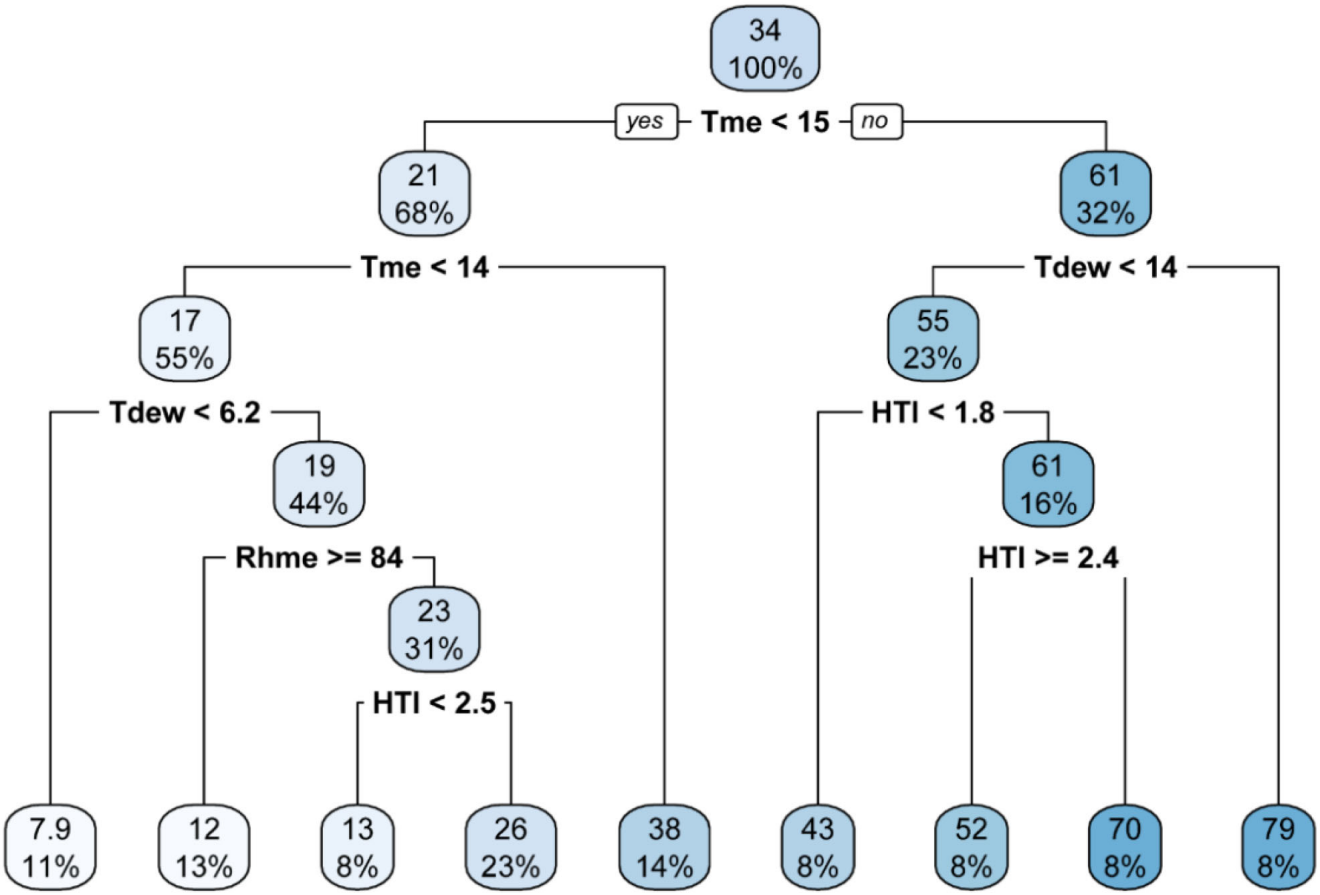
Decision tree with derived weather variable data.

**Figure 9:**
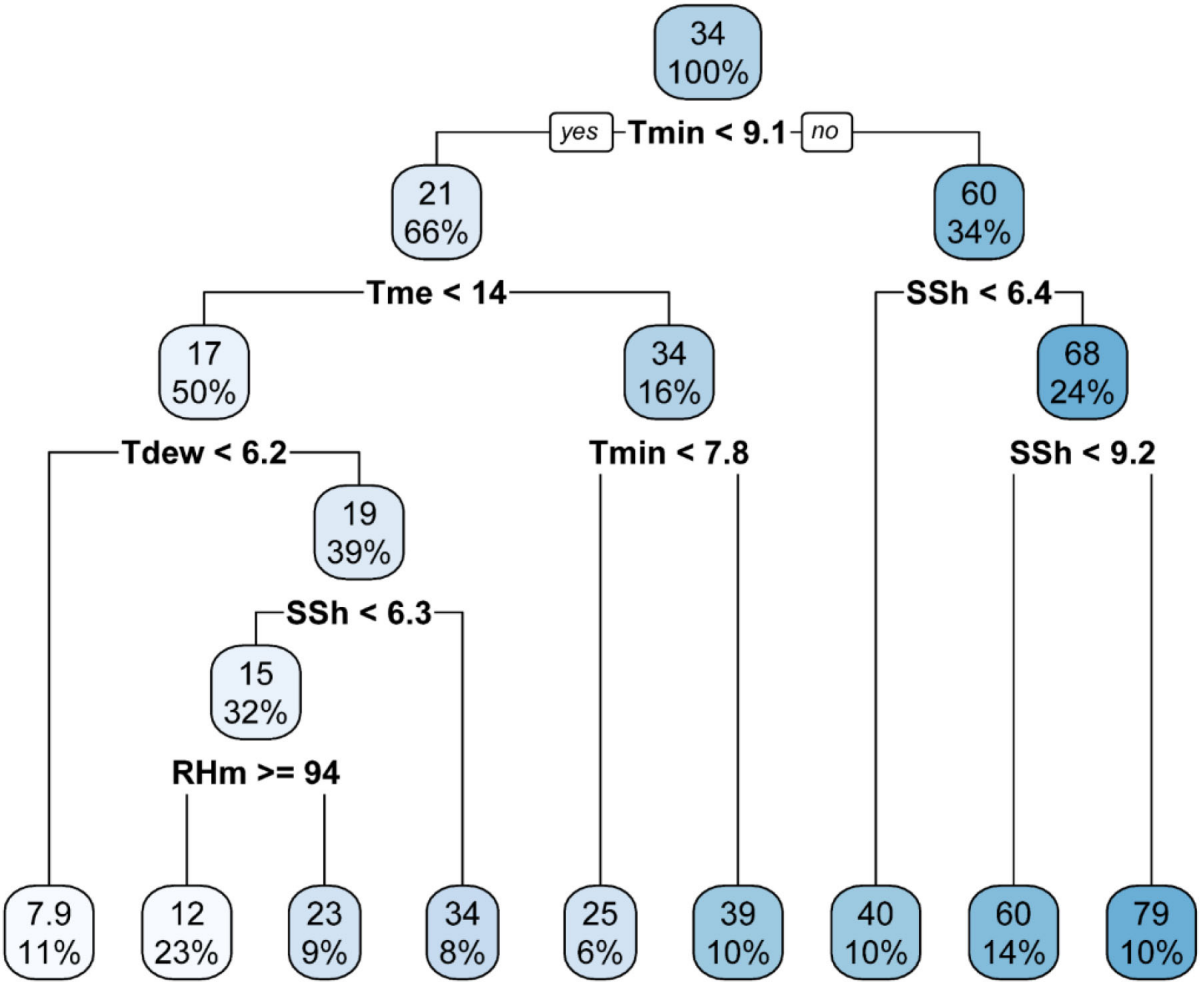
Decision tree with all weather variables data.

Classification and Regression Tree (CART) analysis was used to develop weather-based decision tree models for predicting occurrence of stripe rust. The models were developed using primary, derived and all-weather variables. The CART analysis across primary (**Figure 7**), derived (**Figure 8**), and combined (**Figure 9**) weather variable models consistently highlighted temperature as the dominant factor influencing stripe rust severity. In the primary and all-weather models, maximum severity (79%) was observed when minimum temperature was ≥ 9.1 °C along with sunshine hours ≥ 9.2 h. In the derived variable model, the highest severity (79%) occurred at mean temperature ≥ 15 °C and humid thermal index (HTI) ≥ 2.4. Although both models identified Tmin < 9.1 °C as the root node and showed similar maximum severity (79%) under Tmin ≥ 9.1 °C and SSh ≥ 9.2 h, the combined model provided more refined and biologically meaningful splits under low temperature conditions. Under lower temperature conditions (Tmin < 9.1 °C), disease severity was further governed by interactions among mean temperature, dew point temperature, and relative humidity. Specifically, when Tmean < 14 °C and Tdew < 6.2 °C, severity remained very low (7.9). However, when Tdew ≥ 6.2 °C along with higher relative humidity (RHm ≥ 94%), severity increased to 34 per cent. In contrast, when humidity was relatively lower, severity was restricted to 23 per cent. When Tmean ≥ 14 °C within Tmin < 9.1 °C, severity further increased up to 39 per cent.

### Validation of models

The average magnitude of error between predicted and observed values was measured with help of RMSE. Generally, a lower RMSE indicates a better model. Primary model with higher RMSE of 17.8 indicated more error in predictions as compared to derived (RMSE = 16.0) and all-weather variable (RMSE = 15.2) models (**Table 6**). The primary weather model exhibited R^2^ = 0.70 whereas derived and all-weather variable models exhibited R^2^ = 0.50 and 0.90 respectively. This shows that all weather variable model explained 90% of variation in rust severity. The lowest Relative Root Squared Error (RRSE) of 88.9was exhibited by all-weather variable model as compared to primary (RRSE = 104.4) and derived (RRSE = 93.9) weather variable models. The primary and all-weather models had correlation coefficients of 0.9 and 0.9 unlike derived weather variable model (r = 0.7). Mean absolute error (MAE) was lowest in case of all-weather variable model as compared to primary (MAE = 15.5) and derived weather variable model (MAE = 12.6). The modified index of agreement (D) was high (0.7) in case of all-weather variable model as compared to primary (D = 0.6) and derived (D = 0.6) weather variable models. The m NSE (Modified Nash Sutcliffe Efficiency) of derived and all-weather variable models were 0.2 which shows more predictive performance than primary model (m NSE = -0.04).

**Table 6:** Accuracy metrics of decision tree models.

Model	Variables considered	RMSE	R^2^	RRSE	r	MAE	md Index	m NSE
Primary weather variables model	Tmax, Tmin, RHm, RHe, RF, SSh	17.8	0.7	104.4	0.9	15.5	0.6	-0.04
Derived weather variable model	Tme, RHme, Tdew, HTI	16.0	0.5	93.9	0.7	12.6	0.6	0.2
All weather variable model	All	15.2	0.9	88.9	0.9	12.5	0.7	0.2

## Discussion

The inter-annual variation in yellow rust severity and AUDPC was mainly governed by weather variability during different crop growth periods. The epidemic year 2012–13 coincided with congenial weather conditions that supported rapid spore germination, infection and secondary spread. Conversely, lower disease expression during 2021–22 and 2022–23 was associated with higher temperature and less humid conditions that hampered the infection cycle. The heat wave struck at a critical stage in wheat development i.e. grain filling during 2021-22 (Bal et al., 2022). Temperature exerted the most decisive influence on epidemic development. Both Tmax and Tmin showed strong positive correlations with rust severity and AUDPC, indicating that an optimum thermal window is greatly required for uredospore germination and lesion expansion. Extremely low temperature cause delay in disease onset, whereas higher temperatures during late February reduced disease progression. Latent periods of infection *i.e.* the time between infection and spore generation inside the uredia, can last between 10 and 15 days at 12 to 19°C (Brown et al., 2001). Chen (2005) and Line (2002) also reported that favourable growth and infection at day temperatures between 10-15 °C and night temperatures between 4-8 °C. It has also been observed that a relatively small percentage of spores can travel great distances at high elevations without becoming desiccated (Zeng and Luo, 2006). These could withstand temperatures as low as -10 °C (Gladders et al., 2007). Notably, minimum temperature showed the strongest positive correlation with rust severity highlighting the role of night temperatures in extending leaf wetness duration and increasing nocturnal fungal activity (Sandhu et al., 2021). Minimum disease severity during *rabi* 2021–22 season was marked by daytime and nighttime temperatures up to 35.6 °C and 15.3 °C, respectively which suggests a temperature-induced suppression of rust, consistent with Wellings (2007) and Milus et al. (2009), who found that higher temperatures negatively affect urediniospore viability, germination and infection efficiency. Although newer pathotypes of *Pst* have shown some thermotolerance (Ali et al., 2017), the 2021–22 thermal profile likely exceeded even these adapted thresholds, supporting reduced epidemic potential under heat-stress scenarios. Relative humidity greatly influence disease initiation as well as spread. Morning RH levels were consistently high (91-94%) during the initiation phase, with maximum RH (97%) was observed in epidemic year. However, evening humidity levels, which were substantially lower in 2021-22 (43%) and 2022-23 (46%). Hence, negative correlation was observed between evening relative humidity and rust severity. These results align with De Vallavieille-Pope et al. (1995), who demonstrated that prolonged high humidity especially at night is essential for spore germination and successful infection. Sporulation increases rapidly as relative humidity increases above 50 per cent (Chen et al., 2013). Rainfall contributed to disease spread but was not a primary driver. Moderate rainfall during the progression phase in 2012-13 (22.6 mm) enhanced spore dispersal and canopy wetness, which was consistent with the disease-promoting role of rain splashes and wind-assisted dissemination described by Isard and Russo (2011). However, the overall weak correlation between rainfall and disease implies that rainfall acts as a secondary or complementary factor, enhancing the conducive environment established by temperature and humidity. Moreover, distribution and intensity of rainfall in different disease phases also influences disease progression. Geagea et al. (2000) revealed that rain splashes help uredospores move over short distances. Rains may remove every *Pst* spore from an infected site in about 20 minutes, but it takes nearly 6 hours for that same site to repopulate with an equivalent number of spores. The evaluation of raindrop sizes of 2.5, 3.4, 4.2, and 4.9 mm revealed that larger incident raindrops spread more spores than smaller ones. It was demonstrated by Goldsworthy and Smith (1931) that rust spores need free water to germinate. They demonstrated that germination took place between 5-30°C, with a wide optimal range between 10-28°C, where germination was quick and approached 80 per cent within two hours. The optimal range for germ tube growth was substantially smaller than that for germination, with the most ferocious growth taking place between 15 °C and 20 °C. Germ tube growth also took place between 5 °C and 30 °C. By discovering that light-inhibited hydrated spores may be desensitised by submersion in water, Chang et al. (1974) raised the possibility that a water-soluble inhibitor molecule was at play. The rust fungus may use the increased retardation that goes along with increasing light intensities as an adaptive mechanism to prevent germination on exposed leaf surfaces during the day. When a rainy season is over, these surfaces would be more exposed to the drying effects of the wind and sun than leaf surfaces inside the canopy, putting spores that germinate on them at an increased danger of desiccation and arresting the infection process. Conversely, leaves within the canopy, which require longer or heavier bouts of rain to become wet, would be more readily wetted during rain showers too brief to facilitate infection. While prolonged sunshine hours are generally assumed to reduce leaf wetness and deter disease, a positive correlation was observed with sunshine hours. Anand et al. (2023) demonstrated that optimal urediniospore germination of *Puccinia striiformis* occurred at 15 °C, whereas *Puccinia triticina* peaked at 20 °C, with a neutral pH (7) and intense light (1250 lux) further promoting germination. This anomaly may stem from the role of sunlight in regulating host physiology, such as photosynthesis and stomatal conductance, which can indirectly affect disease susceptibility (Brown et al., 2001). Among derived indices, mean and dew point temperatures were strongly positive factors of rust severity, whereas the humid thermal index (HTI) showed negative relationship, emphasizing the oppressive effect of hot and dry weather. Sandhu et al. (2017) carried out correlation analysis which revealed that maximum and minimum temperature, morning and evening relative humidity, rainfall and sunshine hours influenced the development and progression of the disease. Regression models incorporating multiple weather parameters demonstrated significant R^2^ values for predicting stripe rust occurrence. Stepwise regression analysis indicated that temperature and relative humidity were key indicators for yellow rust prediction. Disease progression was generally slower in December and January but accelerated in February under field conditions. To develop forewarning models for stripe rust in wheat, the relationship between meteorological parameters and disease onset and progress was investigated by Khushboo et al. (2023). An ARIMA (2, 1, 1)(1, 1, 1)7 model with minimum temperature and rainfall as predictors showed 96 per cent accuracy in short-term prediction. Stripe rust severity had a highly significant positive correlation with maximum and minimum temperatures (0.89 and 0.91; 0.91 and 0.75), while morning relative humidity had a significantly negative correlation (-0.84 and -0.80) in different time periods. Rainfall had a non-significant correlation with the disease. Multiple regression models were developed, explaining 91 per cent and 89 per cent of the variation in disease severity, incorporating temperature, relative humidity and rainfall. In Punjab, yellow rust of wheat was shown to have an incubation time and latent duration that varied from 9 to 19 days and 10 to 23 days, respectively, depending on weather factors. Under both natural and artificial situations, polynomial equations were shown to provide the best fit with a significant R^2^ value, which suggested more than 80 per cent variability in disease incidence related to temperature humidity index (Sandhu et al., 2018). The results of PCA are in line with the correlation results. Using Classification and Regression Tree (CART) analysis, decision tree models were developed which conclude that maximum stripe rust severity (79%) can occur when minimum temperature ≥ 9.1 °C and sunshine hours ≥ 9.2 h, or when mean temperature ≥ 15 °C along with humid thermal index (HTI) ≥ 2.4. Yellow rust severity remained moderate (34-39%) when Tmin < 9.1 °C, Tdew ≥ 6.2 °C and RHm ≥ 94%. As per accuracy metrices, these thresholds can be very useful in making crop advisory and developing decision support systems as well. Though many studies have identified most effective meteorological parameter affecting yellow rust severity, but very limited work has been done to quantitatively predict severity of yellow rust that would occur if certain weather conditions prevailed. So, CART analysis effectively removed this gap. Such approach has not previously applied in Punjab. Although previous studies (e.g., Sandhu et al., 2021; Khushboo et al., 2023) have developed predictive models for stripe rust using regression and ARIMA approaches, these models primarily describe statistical relationships and temporal patterns. In contrast, the present study employs CART analysis to derive interpretable, threshold-based decision rules and to capture hierarchical interactions among weather variables. This enhances the operational utility of the model for field-level advisories. Furthermore, the comparative evaluation of primary, derived, and combined weather variables provides additional insights into disease prediction. Also, the present study is based on long-term data from Ludhiana, representing the central plain region of Punjab. As weather–disease interactions are strongly influenced by local agroclimatic conditions, host resistance, and pathogen variability, the derived thresholds and decision tree rules should be considered location-specific. Their direct applicability to other wheat-growing regions of Punjab and the Indo-Gangetic Plains may be limited. Therefore, multi-location validation and recalibration of the model are necessary before wider operational deployment.

## Conclusions

All weather variable model performs best as it has lowest RMSE, highest R^2^ and good performance in most metrics. Primary weather variable model shows strong correlation but falls short in some other variables like MAE and RMSE. Derived weather variable model offers a balanced performance but does not outperform all weather variable model. Together, these decision tree models offer a powerful tool for early disease prediction and proactive management.

## Data Availability

The raw data supporting the conclusions of this article will be made available by the authors, without undue reservation.
